# 

**Published:** 1886-12-20

**Authors:** 


### TABLE OF CONTENTS.

### Original and Selected Articles.

Notes on the Peculiarities of Surgical Practice in the London Hospitals. by Chas. N. Dixon Jones, B.S. M. D....445
Abdominal Surgery, by Arch Dixon....452
Memorial Wreath for the Prosecution in the Graves Malpractice Suit. by L. C. Lane, M.D 456
Treatment of Penetrating Gun-Shot Wounds of the Abdominal Cavity, with Report of Two Cases, by Jos W. Heddens, M. D., St. Joseph, Mo....459
Exudative Retinitis in Bright's Disease....460

### Abstracts and Gleanings.

Retained Placenta....461
Transfusion and Infusion....465
Acute Otorrhœa in Children....468
Delirium Tremens; Menthol....469
Cascara Cordial....470
Diarrhœa in Children; Constipation.... 1
Antiseptic Dressing in Amputation....473
Ingluvin; Sleeplessness; Vinegar as an Antiseptic in the Treatment of Diphtheria....474

### Scientific Items.

A Dog's Reasoning; Parasites of the House Fly.475
Nickel Poison....476

### Practical Notes and Formulæ.

Dysentery; Wizzard Oil....477

### Editorials and Miscellaneous.

Our Journal for 1887; Editorial Brevities; Kind Words....478

Dr. Battey at the Atlanta Society of Medicine..479

The Force of Character....480

Books and Pamphlets Received....482

Special Notices....484

